# Athletic participation and its impact on self-concept, body image, and physical fitness in Saudi female adolescents: a cross-sectional comparison between athletes and non-athletes

**DOI:** 10.7717/peerj.20353

**Published:** 2025-12-03

**Authors:** Asma Alonazi, Deem Almutairi, Taif Alyousef, Afnan Alkhateeb

**Affiliations:** 1 Department of Physical Therapy and Health Rehabilitation, College of Applied Medical Sciences, Majmaah University, Al Majmaah, Riyadh, Saudi Arabia; 2 Department of Physical Therapy, College of Medical Rehabilitation Sciences, King Abdulaziz University, Jeddah, Saudi Arabia

**Keywords:** Self-concept, Self-perception, Body image, Physical fitness, Adolescents, Saudi Arabia, Athletes

## Abstract

**Background:**

Adolescence is a critical developmental period marked by significant physical and psychosocial changes that shape adult behavior and personality. In Saudi Arabia, where female sports participation is newly encouraged, this study therefore aimed to explore how athletic participation influences body image perception, including potential discrepancies between self-perception and fitness outcomes, in a context where female participation in structured physical activity has historically been limited but is now being promoted under Vision 2030.

**Methods:**

A cross-sectional study was conducted in Riyadh province from October 2024 to February 2025, involving 439 female students aged 12–18. Participants were recruited from schools in Al Majma’ah city and were categorized as athletes (school sports participants, *n* = 252) or non-athletes (*n* = 241). Outcomes were assessed using the Physical Self-Inventory-Short (PSI-S) for self-perception, the Stunkard Scale for body image, and physical performance tests (Shuttle Run, V Sit-and-Reach, and Wall Sit Test).

**Results:**

Our findings revealed that athletes reported significantly higher self-perception on the PSI-S compared to non-athletes (53.17% *vs.* 37.34% high self-perception; *p* = 0.002). After body mass index (BMI) adjustment, athletes scored higher on the Stunkard Scale (5.83 *vs.* 4.31; *p* < 0.001). Athletes demonstrated a tendency to perceive their bodies as moderately larger than their actual BMI-adjusted size, a disparity not observed in non-athletes. This self-perception coexisted with superior physical performance, suggesting that athletic training may decouple size perception from functional capability. Athletes outperformed non-athletes in cardiorespiratory fitness (Shuttle Run: 9.96 *vs.* 8.01; *p* < 0.001), flexibility (V Sit-and-Reach: 14.50 *vs.* 13.02; *p* = 0.012), and muscular endurance (Wall Sit Test: 63.17 *vs.* 58.30; *p* = 0.024). No significant differences in self-perception were observed across BMI categories, suggesting athletic participation’s benefits transcend weight status.

**Conclusion:**

The findings align with global research linking physical activity to enhanced body satisfaction and self-worth. However, athletes’ self-perception of larger body figures may reflect societal pressures toward thinness, highlighting the complex interplay of cultural ideals and body image. The study underscores the role of sports in promoting physical fitness and psychological well-being among Saudi adolescent females, supporting national efforts to encourage female athletic participation.

## Introduction

Adolescence is a critical developmental stage characterized by heightened bodily awareness, identity formation, and self-concept development (Jaworska & MacQueen, 2015). Females in this phase often become particularly sensitive to their appearance, influenced by sociocultural, familial, and peer dynamics (Song et al., 2023). Body image, a perceptual, cognitive, and emotional evaluation of one’s body, plays a vital role in shaping self-concept and psychological well-being (Bodega et al., 2023; Toselli et al., 2023). Unrealistic societal expectations can lead to distorted body image, resulting in negative health outcomes such as disordered eating, low self-esteem, and emotional distress (Schuck, Munsch & Schneider, 2018). According to Festinger’s Social Comparison Theory developed in 1954, adolescents often evaluate their bodies by comparing themselves to peers or media ideals. For female athletes, this may create tension between the physical demands of athletic performance and the societal ideal of thinness, a dynamic particularly understudied in Saudi contexts, where female participation in sports is only recently gaining societal acceptance. Regular physical activity may, however, offer a positive influence on self-concept and body image, providing a pathway to explore how athletic participation shapes these outcomes.

Physical activity is a significant factor in this context. Research data indicate low physical activity among adolescents, females in particular, who consistently demonstrate inactive lifestyle (Manzano-Sánchez et al., 2022). However, adolescents who participate in sports activities exhibit relatively lesser body dissatisfaction, likely due to lowering adiposity and, subsequently, lesser body dissatisfaction (Leone et al., 2025; Tebar et al., 2021).

In Saudi Arabia, female participation in structured physical activity has historically been limited, with formal physical education for females only introduced in 2017 (Al Ruwaili, 2020). This policy shift aligned with Vision 2030, a national initiative promoting women’s empowerment and public health. With these reforms, understanding how sports engagement influences Saudi adolescent females’ perceptions of self and body image becomes both timely and essential.

Vision 2030s Quality of Life Program explicitly prioritizes female sports participation, with initiatives like school sports leagues and community fitness centers. This policy shift aims to increase females’ physical activity rates from 13% to 40% by 2030 (Quality of Life Program, 2016), yet its psychosocial impacts remain unexplored.

Studies suggest that physical activity enhances body satisfaction and self-worth, potentially mitigating risks of low self-esteem, disordered eating, and mood disturbances (Andermo et al., 2020; Lee & Lee, 2016; Liu, Menhas & Saqib, 2024; Melo et al., 2025; Thorup et al., 2024). This suggests the importance of dissecting physical activity and particularly sports participation among middle and high school female students and whether it influences the perception of their physical self and perceived body figure.

However, the cultural context within Saudi Arabia provides a distinctive perspective for exploring these associations. Therefore, this study aims to compare self-concept and body image between Saudi adolescent female athletes and non-athletes, and to evaluate differences in physical performance indicators, including cardiorespiratory fitness, flexibility, muscular endurance. Additionally, this study examines the influence of athletic participation on self-perception and body image in a cultural context where female sports participation is newly encouraged and evolved.

## Materials & Methods

### Ethics considerations

Ethical approval was obtained from Majmaah University Institutional Review Board (Reference # MUREC-Mar.3/COM-2024/7-1). Written informed consents were secured from parents, and a signed assent from students. The anonymity and confidentiality of the participants were protected throughout the study.

### Study design and participants

A cross-sectional study design was conducted between October 2024 and February 2025 in Al Majma’ah, Riyadh Province. Included 493 female students, divided nearly equally between athletes (*n* = 252) and non-athletes (*n* = 241). Although a formal *a priori* power analysis was not conducted, this sample size is adequate for detecting medium effect sizes with 80% power at a significance level of 0.05, as supported by standard guidelines for *t*-tests and chi-square analyses in cross-sectional designs.

Female students aged 12–18 was recruited from middle and high schools and categorized as either athletes (participating in school official sports club) or non-athletes. Healthy female students aged 12–18 years were recruited. Schools were randomly selected. Males, individuals with neurological or musculoskeletal conditions, and those unwilling to participate were excluded.

### Outcome measures

• **Physical Self-Inventory-Short (PSI-S)**: The PSI-S evaluates multidimensional aspects of physical self-concept, including perceived strength, attractiveness, and overall physical self-worth. It is a validated tool that captures adolescents’ internal evaluations of their physical capabilities and image, directly aligning with the study’s focus on self-perception. It uses a Likert-type scale, ranging from a 6-point Likert scale (1 = Not at all to 6 = Entirely). Overall, scores are categorized into low (18–36), moderate (54–72), and high (90–108). Low scores suggest poor self-perception and possible dissatisfaction, moderate scores suggest neutral perception, neither positive nor negative, and high scores indicate stronger positive perception (Morin et al., 2016).
• **Stunkard Scale**: This visual scale assesses perceived body image through silhouette selection. It helps quantify body dissatisfaction by comparing a participant’s self-identified body shape with culturally internalized ideals. This tool is particularly useful in adolescent populations where visual body cues heavily influence self-concept. It requires participants to select the figure corresponding with their self-perception of current body type or body type they believe is standard (Stunkard, Sørensen & Schulsinger, 1983).
• **Shuttle Run Test**: Chosen to assess cardiorespiratory fitness and agility, the shuttle run reflects physical performance, which may impact one’s self-concept and perceived competence. Better performance can be linked to higher self-worth and body satisfaction. It requires participants to run between two cones which are placed 20 m apart (Cadenas-Sánchez et al., 2014).
• **V-Sit-and-Reach** **Test:** This test measures flexibility, which is often associated with physical capability in sports. Improvements in flexibility contribute to a sense of physical competence and can influence how adolescents perceive their functional abilities and body (Das et al., 2022). It requires participant to sit on the floor with extended legs and leaning forward with both hands towards the toes.
• **Wall Sit Test**: The wall sit test evaluates lower limb muscular endurance, a key indicator of athletic fitness. Stronger performance in this test supports a more positive perception of physical strength, contributing to better physical self-concept and body confidence. It requires participant to stay in a sitting posture while leaning back against a wall with bent knee at 90° and time is recorded (Philippe et al., 2022; Tomchuk, 2011).

### Study procedure and data collection

The director of each school in Riyadh province was contacted and informed about the objectives of the study. An official written permission letter was submitted to the school administrations to obtain approval for conducting the research. Upon receiving formal written authorization, the research team coordinated with a designated teaching faculty member at each school. Prior to student enrollment, informed consent forms were distributed through the teaching faculty to students and their parents. Ample time was provided for parents to review the forms, ask relevant questions, and make an informed decision before signing.

Following parental consent during the initial visit, the research team identified athletes by directly approaching female students who were actively participating in school sports teams or athletic clubs. The aims and procedures of the study were thoroughly explained, and verbal assent was obtained before beginning any measurements. For the non-athlete group, students were selected based on teacher recommendations confirming that they were not enrolled in any school or extracurricular sports programs and did not engage in regular physical activity in any form.

Subsequently, research team members demonstrated each test and ensured appropriate completion of the Physical Self-Inventory–Short (PSI-S), Stunkard Scale, Shuttle Run Test, V Sit-and-Reach Test, and Wall Sit Test. To minimize fatigue and ensure reliable performance, participants were given a two-minute rest period between each test.

### Statistical analyses

Data entry and management were conducted using Office 365’s Microsoft Excel, while the data analysis was performed using the Stata 18 software (StataCorp. 2023. Stata Statistical Software: Release 18. College Station, TX: StataCorp LLC). Quantitative data were presented as means ± standard deviations (SDs) and categorical data were shown as frequencies and percentages. Chi-square (*χ*^2^) test was applied to evaluate the difference of PSI-S self-perception categories (low, moderate, and high) between non-athletes and athletes. In addition, stratified analyses were conducted by stratifying participants based on body mass index (BMI) categories (normal, underweight, overweight, and obese) to investigate whether the differences varied across BMI groups. To determine mean differences for Stunkard Scale, Shuttle Run Test, V Sit-and-Reach Test, and Wall Sit Test between non-athletes and athletes, an independent samples *t*-test was conducted. Adjusted mean values (adjusted for BMI (Kg/m^2^)) for aforementioned tests along with 95% confidence intervals (CIs) were obtained by applying analysis of covariance (ANCOVA). The *p* value <0.05 was considered statistically significant.

## Results

### Demographic characteristics of study participants

In the present research study, a total of 493 female school students consented and participated. The distribution of participants based on their athletic status was almost identical; non-athletes (*n* = 241, 48.9%) and athletes (*n* = 252, 51.1%). In anthropometric measurement, mean BMI was 24.66 ± 6.78 kg/m^2^. On PSI-S, 101 (20.5%) participants had low, 168 (34.1%) had moderate, and 224 (45.4%) high self-perception. Mean scores on Stunkard Scale, Shuttle Run Test, V Sit-and-Reach Test, and Wall Sit Test were 5.09 ± 2.29, 9.01 ± 1.55, 13.77 ± 6.59, and 60.79 ± 23.86, respectively. Table 1 demonstrates the demographic characteristics middle and high school female non-athletes and athletes.

**Table 1:** Demographic characteristics of middle and high school female non-athletes and athletes (*n* = 493).

**Characteristics**	**Frequency (%)**
Age, years (Mean ± SD)	16.88 ± 1.27
Sports-based participant distribution	
Non-athletes	241 (48.9%)
Athletes	252 (51.1%)
Anthropometric measurements	
Height, cm (mean ± SD)	159.69 ± 7.09
Weight, kg (mean ± SD)	62.79 ± 17.15
BMI, kg/m^2^ (mean ± SD)	24.66 ± 6.78
Outcome measures	
PSI-S	
*Low self-perception*	101 (20.5%)
*Moderate self-perception*	168 (34.1%)
*High self-perception*	224 (45.4%)
Stunkard scale (Mean ± SD)	5.09 ± 2.29
Shuttle run test (Mean ± SD)	9.01 ± 1.55
V sit-and-reach test (Mean ± SD)	13.77 ± 6.59
Wall sit test (Mean ± SD)	60.79 ± 23.86

**Notes.**

Abbreviations SD Standard Deviation BMI Body Mass Index PSI-S Physical Self-Inventory–Short

### Comparison of self-perception (PSI-S) between non-athletes and athletes

Table 2 presents the comparison of self-perception using PSI-S questionnaire between non-athletes and athletes with stratified analysis by BMI categories. Overall, fairly higher proportion of athletes demonstrated high self-perception in contrast to non-athletes (53.17% *versus* 37.34%). Similarly, compared to non-athletes, low (17.46% *versus* 23.65%) and moderate (29.37% *versus* 39.00%) self-perception was noted lesser among athletic female students. The overall findings were statistically significant (*p* = 0.002). When stratified analysis was performed based on BMI categories, no statistical difference was observed between non-athletes and athletes for self-perception (normal; *p* = 0.170, underweight; *p* = 0.326, overweight; *p* = 0.111, and obese; *p* = 0.390).

**Table 2:** Comparison of self-perception (PSI-S) between non-athletes and athletes, stratified by BMI categories.

**BMI category**	**Athletic status**	**Low PSI-S***n*(%)	**Moderate PSI-S***n* (%)	**High PSI-S***n* (%)	***p*** ** value**
Overall	Non-athletes	57 (23.65%)	94 (39.00%)	90 (37.34%)	0.002
	Athletes	44 (17.46%)	74 (29.37%)	134 (53.17%)	
Normal	Non-athletes	10 (12.35%)	30 (37.04%)	41 (50.62%)	0.170
	Athletes	9 (9.38%)	25 (26.04%)	62 (64.58%)	
Underweight	Non-Athletes	12 (23.53%)	14 (27.45%)	25 (49.02%)	0.326
	Athletes	8 (14.81%)	12 (22.22%)	34 (62.96%)	
Overweight	Non-Athletes	14 (29.79%)	23 (48.94%)	10 (21.28%)	0.111
	Athletes	11 (23.91%)	16 (34.78%)	19 (41.30%)	
Obese	Non-Athletes	21 (33.87%)	27 (43.55%)	14 (22.58%)	0.390
	Athletes	16 (28.57%)	21 (37.50%)	19 (33.93%)	

**Notes.**

Abbreviations PSI-S Physical Self-Inventory- Short BMI Body Mass Index

### Mean differences for stunkard scale, shuttle run test, V sit-and-reach test, and wall sit test between non-athletes and athletes

Mean values of instruments assessing perceived body figure (Stunkard Scale), cardiorespiratory fitness (Shuttle Run Test), lower back and hamstring muscles flexibility (V Sit-and-Reach Test), and lower body strength and muscular endurance (Wall Sit Test) are presented in Table 3. Mean scores on instruments were significantly higher for athletic female students middle and high schools in comparison to non-athletes; Stunkard Scale (5.81 ± 2.10 *versus* 4.34 ± 2.24; *p* < 0.001), Shuttle Run Test (9.96 ± 1.19 *versus* 8.01 ± 1.22; *p* < 0.001), V Sit-and-Reach Test (14.53 ± 7.49 *versus* 12.98 ± 5.41; *p* = 0.009), and Wall Sit Test (63.20 ± 24.14 *versus* 58.27 ± 23.36; *p* = 0.022).

**Table 3:** Mean values of Stunkard scale, shuttle run test, V sit-and-reach test, and wall sit test in the study groups according to athletic status.

**Variables**	**Athletic Status**		***p*** ** Value**
	**Non-Athletes** **(*n* = 241)**	**Athletes** **(*n* = 252)**	
Stunkard scale	4.34 ± 2.24	5.81 ± 2.10	<0.001
Shuttle run test	8.01 ± 1.22	9.96 ± 1.19	<0.001
V sit-and-reach test	12.98 ± 5.41	14.53 ± 7.49	0.009
Wall sit test	58.27 ± 23.36	63.20 ± 24.14	0.022

**Notes.**

Abbreviation CI Confidence Interval

Data are presented as mean ± standard deviation.

**Figure 1:**
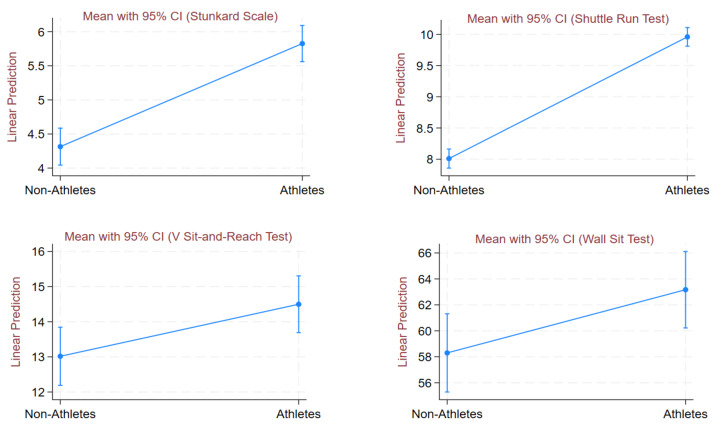
Margins plot for BMI-adjusted mean (Stunkard Scale, Shuttle run test, V sit-and-reach test, and wall sit test) for athletes and non-athletes.

Adjusted mean values of instruments are shown in Table 4. Data were adjusted for a BMI (Kg/m^2^) covariate. After BMI adjustment, the findings remained statistically significant and revealed that athletes had significantly higher mean scores on Stunkard Scale (5.83 *versus* 4.31; *p* < 0.001), Shuttle Run Test (9.96 *versus* 8.01; *p* < 0.001), V Sit-and-Reach Test (14.50 *versus* 113.02; *p* = 0.012), and Wall Sit Test (63.17 *versus* 58.30; *p* = 0.024) compared to non-athletes. Figure 1 displays margins plot for BMI-adjusted mean and 95% CI for Stunkard Scale, Shuttle Run Test, V Sit-and-Reach Test, and Wall Sit Test between non-athletes and athletes.

**Table 4:** Adjusted mean values of Stunkard scale, shuttle run test, V sit-and-reach test, and wall sit test (95% CI) in the study groups according to athletic status.

**Variables**	**Athletic status**		***p*** ** value**
	**Non-Athletes** **(*n* = 241)**	**Athletes** **(*n* = 252)**	
Stunkard scale	4.31 (4.04–4.59)	5.83 (5.56–6.09)	<0.001
Shuttle run test	8.01 (7.86–8.16)	9.96 (9.81–10.11)	<0.001
V sit-and-reach test	13.02 (12.19–13.84)	14.50 (13.69–15.31)	0.012
Wall Sit Test	58.30 (55.29–61.31)	63.17 (60.22–66.11)	0.024

**Notes.**

Abbreviation CI Confidence Interval

Data are presented as mean (95% CI). The model was adjusted for BMI (Kg/m^2^).

## Discussion

Adolescence is an extremely sensitive age bracket which determines the development and progression of self-perception and body image over years (Doğan et al., 2018). They are at the receiving end of a vast number of cues implying what is socially likeable and objectionable, separating them from realism during their developmental years. There is a complex relation among the ubiquitous exposure to social media, cultural norms, self-perception and body image satisfaction, and its considerable psychological repercussions linked with so called societal ‘beauty standards’ (Merino et al., 2024). One factor that has been recently cited to improve the self-perception and body image satisfaction concerns among adolescents is physical activity (Gualdi-Russo, Rinaldo & Zaccagni, 2022). Getting insight of the self-perception and body image landscape among school-going adolescents females through the lens of athleticism is crucial for understanding its merit and encouraging physical sports and similar interventions from young age for psychological and mental well-being.

The findings of the present study suggested that school-going adolescent female athletes may have better perception of themselves regarding self-image and physical health and attractiveness compared to that of non-athletic females. An earlier research study communicated that girls with increased physical activity are more likely to have increased social self-perception and higher athletic self-perception (Stein et al., 2007). A systematic review by Gualdi-Russo, Rinaldo & Zaccagni (2022) reported physical activity as the protective factor for body image perception and satisfaction among adolescents (Gualdi-Russo, Rinaldo & Zaccagni, 2022). The reason why physically active adolescents may experience higher self-perception, positive body image, and satisfaction is because of their athletic bodies that match cultural norms and societal acceptance. Therefore, athleticism may put them at lower risk of self-image and self-perception issues owing to their bodies resembling the body image as outlined and approved in the society (Burgon, Beard & Waller, 2023).

While males can get affected by the issues of body image and self-perception, the problem remains prevalent in females (Latiff, Muhamad & Rahman, 2018). In the present study, we only enrolled female adolescents though. A study by Pinho et al. (2019) demonstrated that dissatisfaction with body image and concerns related to body weight are predominantly exhibited by females in contrast to males (Pinho et al., 2019). Adolescent females are more likely than boys to focus on their physical aesthetics on social media and are subject to body image associated pressure, dissatisfaction, and self-condemnation (Mahon & Hevey, 2021). Interestingly, an older research from Australia found adolescent boys to be more inclined towards investing in functional behavior and functional satisfaction than aesthetic value and satisfaction (Abbott & Barber, 2010).

Notably, female athletes perceived themselves as moderately larger on the Stunkard Scale, reflecting societal pressures toward thinness (Tort-Nasarre, Pollina Pocallet & Artigues-Barberà, 2021). Even within healthy anthropometric ranges, they may overestimate their size due to aspirational thinness ideals. This could stem from competing standards: athletic training increases muscle mass (Zaccagni & Gualdi-Russo, 2023), yet Social Comparison Theory (Festinger, 1954) suggests women also internalize societal thinness ideals (Burgon, Beard & Waller, 2023). In Saudi Arabia’s evolving cultural landscape, this dual comparison may uniquely distort self-perception. Future studies should integrate objective measures (*e.g.*, DEXA scans) to disentangle these effects.

Athletic females had fairly superior cardiorespiratory fitness, lower back and hamstring muscles flexibility, and lower body strength and muscular endurance, highlighting the significance of sports in overall physical fitness. The World Health Organization (WHO) guidelines (2021) endorse *‘children and adolescents should do at least an average of 60 min per day of moderate- to vigorous-intensity, mostly aerobic, physical activity, across the week’* and *‘vigorous-intensity aerobic activities, as well as those that strengthen muscle and bone, should be incorporated at least 3 days a week’* (Bull et al., 2020). However, data suggests no improvement in physical activity at a worldwide level between 2001 and 2016 (Guthold et al., 2018) and 81% of adolescents do not even adhere to aerobic exercise guidelines (Guthold et al., 2020). It has been reported that involvement in physical activity improves physical, mental, and psychosocial health which eventually benefits academics and cognition (Ardoy et al., 2014). Physical education or school-related interventions can be used to enhance physical involvement and performance among adolescents (Burke et al., 2014). It is documented that peer-driven knowledge regarding physical activity could help encourage physical activity among adolescent girls (Sebire et al., 2019).

### Strengths and limitations

This study has several limitations that should be acknowledged. First, its cross-sectional design restricts the ability to infer causality between athletic participation and self-perception or body image. Second, data on athletic status and physical self-perception were based on self-report, which may be influenced by social desirability bias, particularly in adolescent girls within conservative settings. Third, the Stunkard Scale silhouettes may not accurately reflect the diverse body types of Saudi adolescents, potentially affecting validity. Fourth, Lack of dietary/nutritional data, which may confound body image perceptions. Furthermore, although the Stunkard scale is a widely used tool for assessing body image perception, it has not been formally validated in the Saudi population. Future studies are encouraged to culturally adapt and validate this instrument to ensure its appropriateness in the local context. Although BMI was included and adjusted for in our analyses, we did not specifically evaluate the concordance between participants’ actual BMI and the figure selected as ‘feel’. Future research should address this gap to better assess the accuracy of body image perception among adolescents. Lastly, the study was geographically limited to Al Majma’ah City, reducing the generalizability of findings to other regions of Saudi Arabia.

### Recommendations

We believe that following recommendations could work as a catalyst for future research work in this domain: (1) we encourage that future work should focus on utilizing more objective measures of body size and shape in school-going Saudi female adolescents like that undertaken by Speight (2013), (2) it is equally important for males to be enrolled along with females to understand the gender-based differences of involvement in physical activity, self-concept, and body image, (3) participants from other geographical regions in Saudi Arabia must be included as this would enhance the generalization of the research findings, (4) longitudinal designs to track changes post-sports participation, and (5) a qualitative or mixed method study can be conducted to gain precise understanding of the sociocultural influence and adolescents’ self-perception.

## Conclusions

The current study concludes that school-going adolescent female athletes may have better perception of themselves regarding self-image and physical health and attractiveness compared to that of non-athletic females. However, athletes perceived themselves as somewhat overweight or having moderately larger body as evidenced by average self-rating on Stunkard Scale with body image silhouettes. Athletic females had fairly superior cardiorespiratory fitness, lower back and hamstring muscles flexibility, and lower body strength and muscular endurance, highlighting the significance of sports in overall physical fitness. Prospective investigators should focus on more objective measures such as 3D body imaging for body size and shape to account for differences in perceived body image and actual body image among adolescents. Finally, policy makers, stakeholders, and school administrators should address the importance of physical health and active lifestyle among adolescents in Saudi Arabia beyond its role in attractiveness and as an indispensable component of mental strength, quality of life, and overall health and well-being.

##  Supplemental Information

10.7717/peerj.20353/supp-1 Supplemental Information 1 Raw data

## Additional Information and Declarations

### Data Availability

The following information was supplied regarding data availability: The raw data is available in the Supplementary File.
